# Supplementary material for: Multi‐dataset identification of innovative feature genes and molecular mechanisms in keratoconus
Source: J Cell Mol Med. 2024 Sep 19;28(18):e70079. doi: 10.1111/jcmm.70079 (PMC11412914; doi:10.1111/jcmm.70079)
Supplement: Supplementary file 2 — Material S2. [file JCMM-28-e70079-s002.zip › 7.GSEA analysis/4.MYRF/KEGG_BASAL_TRANSCRIPTION_FACTORS.html]

Details for gene set KEGG\_BASAL\_TRANSCRIPTION\_FACTORS[GSEA]

|  || Dataset | MYRF.group.cls#h\_versus\_l |
| Phenotype | group.cls#h\_versus\_l |
| Upregulated in class | l |
| GeneSet | KEGG\_BASAL\_TRANSCRIPTION\_FACTORS |
| Enrichment Score (ES) | -0.32912123 |
| Normalized Enrichment Score (NES) | -1.40358 |
| Nominal p-value | 0.1199187 |
| FDR q-value | 0.23710085 |
| FWER p-Value | 0.879 |
Table: GSEA Results Summary

  

Fig 1: Enrichment plot: KEGG\_BASAL\_TRANSCRIPTION\_FACTORS      
 Profile of the Running ES Score & Positions of GeneSet Members on the Rank Ordered List

  

| SYMBOL | TITLE | RANK IN GENE LIST | RANK METRIC SCORE | RUNNING ES | CORE ENRICHMENT || 1 | TBPL2 | na | 5612 | 0.205 | -0.1034 | No |
| 2 | GTF2A1 | na | 7853 | 0.142 | -0.1275 | No |
| 3 | TAF5L | na | 8600 | 0.125 | -0.1135 | No |
| 4 | TAF1 | na | 10012 | 0.096 | -0.1268 | No |
| 5 | GTF2E1 | na | 10629 | 0.084 | -0.1205 | No |
| 6 | STON1 | na | 12374 | 0.058 | -0.1542 | No |
| 7 | GTF2E2 | na | 12593 | 0.055 | -0.1446 | No |
| 8 | TAF6L | na | 12980 | 0.051 | -0.1413 | No |
| 9 | TAF1L | na | 13500 | 0.044 | -0.1438 | No |
| 10 | GTF2H4 | na | 15087 | 0.027 | -0.1818 | No |
| 11 | GTF2H1 | na | 15739 | 0.020 | -0.1948 | No |
| 12 | GTF2I | na | 16560 | 0.012 | -0.2149 | No |
| 13 | TAF13 | na | 17708 | 0.002 | -0.2473 | No |
| 14 | TAF2 | na | 19549 | -0.017 | -0.2956 | No |
| 15 | TAF12 | na | 20449 | -0.027 | -0.3138 | No |
| 16 | TAF6 | na | 20860 | -0.032 | -0.3165 | Yes |
| 17 | GTF2A2 | na | 21144 | -0.035 | -0.3146 | Yes |
| 18 | GTF2F2 | na | 21583 | -0.041 | -0.3157 | Yes |
| 19 | TAF4 | na | 22050 | -0.046 | -0.3159 | Yes |
| 20 | TBP | na | 22371 | -0.050 | -0.3107 | Yes |
| 21 | TBPL1 | na | 22745 | -0.056 | -0.3056 | Yes |
| 22 | TAF9 | na | 22904 | -0.058 | -0.2936 | Yes |
| 23 | TAF7 | na | 23240 | -0.063 | -0.2852 | Yes |
| 24 | GTF2H3 | na | 23444 | -0.066 | -0.2721 | Yes |
| 25 | GTF2B | na | 23447 | -0.066 | -0.2532 | Yes |
| 26 | TAF11 | na | 23903 | -0.074 | -0.2452 | Yes |
| 27 | GTF2F1 | na | 24109 | -0.078 | -0.2289 | Yes |
| 28 | TAF10 | na | 25995 | -0.113 | -0.2511 | Yes |
| 29 | TAF9B | na | 27232 | -0.140 | -0.2467 | Yes |
| 30 | GTF2IRD1 | na | 29187 | -0.197 | -0.2469 | Yes |
| 31 | TAF5 | na | 31315 | -0.281 | -0.2282 | Yes |
| 32 | TAF4B | na | 32311 | -0.337 | -0.1607 | Yes |
| 33 | TAF7L | na | 34642 | -0.807 | 0.0025 | Yes |
Table: GSEA details [plain text format]

  

Fig 2: KEGG\_BASAL\_TRANSCRIPTION\_FACTORS      
 Blue-Pink O' Gram in the Space of the Analyzed GeneSet

  

Fig 3: KEGG\_BASAL\_TRANSCRIPTION\_FACTORS: Random ES distribution      
 Gene set null distribution of ES for **KEGG\_BASAL\_TRANSCRIPTION\_FACTORS**
